
JOURNAL INFORMATION
==============================
NLM Title Abbreviation: Hum Brain Mapp
Journal ID: Hum Brain Mapp
NLM Journal ID: 9419065
Journal ID: HBM

Human Brain Mapping

ISSN: 1065-9471
EISSN: 1097-0193
Publisher: Wiley

ARTICLE INFORMATION
==============================
PMCID: PMC11474691
DOI: 10.1002/hbm.25940
Article ID: HBM25940
Article version: 1
Subjects: Issue Information, Issue Information

Editorial board ‐ TOC

Electronic publication date: 2023 Nov 1
Publication date: 2023 Dec 1
Volume: 44
Issue: 17
First page: 5515
Last page: 5522
Copyright: © 2023 The Authors. Human Brain Mapping published by Wiley Periodicals LLC.
License: This is an open access article under the terms of the http://creativecommons.org/licenses/by/4.0/ License, which permits use, distribution and reproduction in any medium, provided the original work is properly cited.
License URL: https://creativecommons.org/licenses/by/4.0/

Figure count: 0
Table count: 0
Page count: 6

source-schema-version-number: 2.0
cover-date: 1 December, 2023
details-of-publishers-convertor: Converter:WILEY_ML3GV2_TO_JATSPMC version:6.4.9 mode:remove_FC converted:15.10.2024

==============================

### HUMAN BRAIN MAPPING

Open Access

Volume 44, Issue 17, 1 December, 2023

View this journal online at wileyonlinelibrary.com

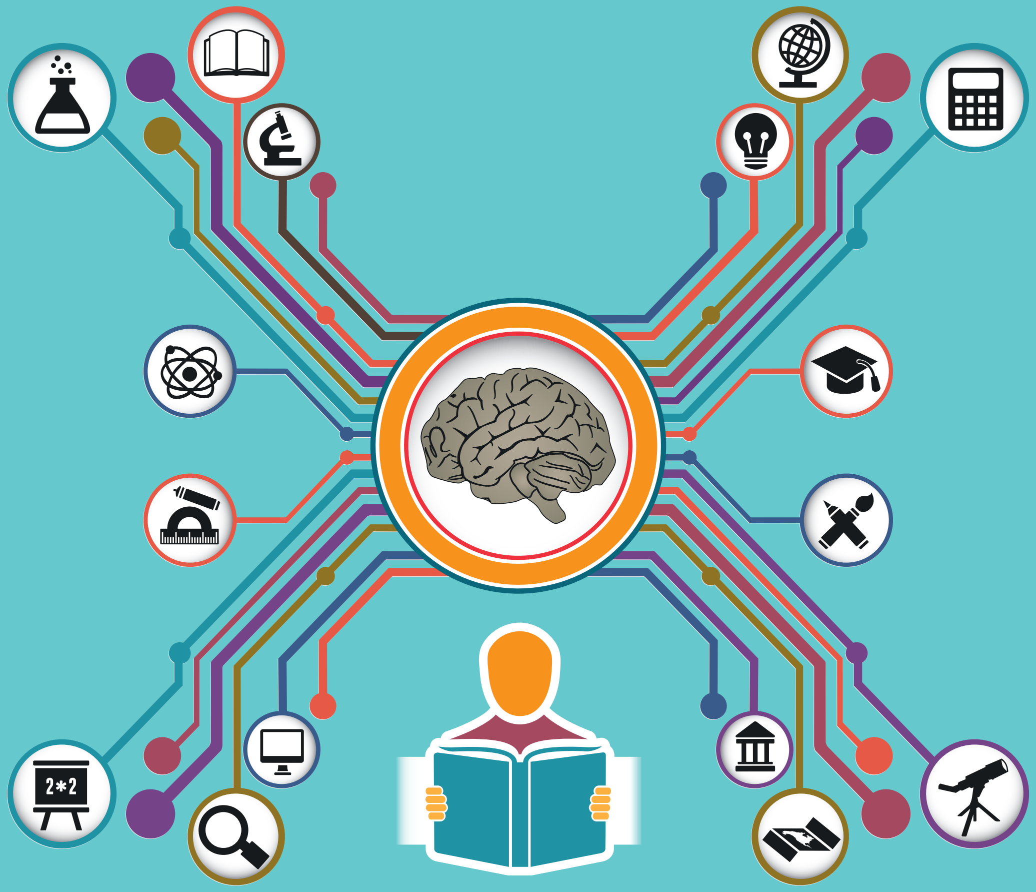

WILEY

ISSN 1097-0193

### HUMAN BRAIN MAPPING

### A Journal Devoted to Functional Neuroanatomy and Neuroimaging

### *Editor-in-Chief*

**Simon B. Eickhoff**
Institute for Systems Neuroscience
Heinrich-Heine University, Düsseldorf, Germany

### *Deputy Editors-in-Chief*

**Gary Egan**
Monash Biomedical Imaging
Monash University, Victoria, Australia

**Sophia Frangou**
Icahn School of Medicine
at Mount Sinai, New York, USA

**Martijn van den Heuvel**
Brain Center Rudolf Magnus,
University of Medical Center,
Utrecht, Netherlands

### *Associate Editors*

**Annabel Chen**
Nanyang Technological University, Singapore

**Michael J. Farrell**
Monash University, Victoria, Australia

**Massimo Filippi**
Università Vita-Salute San Raffaele, Milan,
Italy

**Sophia Frangou**
Icahn School of Medicine at Mount Sinai
New York, USA

**Cheryl L. Grady**
Rotman Research Institute
Toronto, Canada

**Joachim Gross**
Centre for Cognitive Neuroimaging
University of Glasgow, Glasgow, UK

**Martin A. Lindquist**
John Hopkins, Bloomberg School of Public
Health, MD, USA

**Tomáš Paus**
Rotman Research Institute
Toronto, Canada

**Indira Tendolkar**
Radboud University, Nijmegen,
The Netherlands

**Paul Thompson**
Laboratory of Neuro Imaging,
University of Southern California,
Los Angeles, CA, USA

**Helen Zhou**
Yong Loo Lin School of Medicine, National
University of Singapore, Singapore

### *Editorial Board*

**Susan Bookheimer**
Center for Cognitive Neuroscience
UCLA School of Medicine, USA

**Allen R. Braun**
NIDCD, National Institutes of Health
Bethesda, USA

**Christian Buechel**
Univ-Krankenhaus Eppendorf
Hamburg, Germany

**Marco Catani**
King's College London, UK

**Franco Cauda**
University of Turin, Italy

**Vincent Clark**
University of New Mexico, USA

**Olivier David**
Université Joseph Fourier, France

**Mark D'Esposito**
University of California, Berkeley,
USA

**Julien Doyon**
Functional Neuroimaging Unit
University of Montreal, Canada

**Eugene Duff**
University of Oxford, UK

**Timothy Q. Duong**
Research Imaging Institute,
University of Texas Health Science
Center, USA

**Paul Fletcher**
University of Cambridge, UK

**Jia-Hong Gao**
Brain Research Imaging
Center The University of Chicago,
USA

**David C. Glahn**
Yale University School of Medicine
Hartford, USA

**Gregory S. Hickok**
Center for Cognitive
Neuroscience University of California,
Irvine, USA

**Barry Horwitz**
NIDCD, National Institutes of Health
USA

**Peter Jezzard**
John Radcliffe Hospital
University of Oxford, UK

**David N. Kennedy**
Harvard Medical School, USA

**Neda Ladbon-Bernasconi**
Montreal Neurological Institute
Canada

**Nicholas Lange**
Harvard Medical School, USA

**Tatia M. C. Lee**
State Key Laboratory of Brain &
Cognitive Sciences, The University of
Hong Kong

**Louis Lemieux**
UCL Institute of Neurology, UK

**Bernard Mazoyer**
CNRS/CEA, Université de Bordeaux
France

**Gregory McCarthy**
Yale University, USA

**A. Randy McIntosh**
Rotman Research Institute
Toronto, Canada

**Janine Mendola**
McGill Vision Research
McGill University, Canada

**Thomas Nichols**
University of Warwick, UK

**William Penny**
Wellcome Trust Centre for
Neuroimaging, UCL Institute of
Neurology, UK

**Laurent Petit**
CNRS, Université de Bordeaux,
France

**Russel A. Poldrack**
Stanford Unversity, USA

**Jean-Baptiste Poline**
Imagen Orsay Service Hospitalier
Frédéric Joliot, France

**Stephen Smith**
Center for Functional MRI of the
Brain, University of Oxford, UK

**Elizabeth Sowell**
Children'sHospital Los Angeles, USA

**Li Hai Tan**
Shenzhen Institute of Neuroscience
Shenzhen, P.R. China

**Kate Watkins**
John Radcliffe Hospital
University of Oxford, UK

**Richard G. Wise**
Cardiff University, UK

**Roger Woods**
UCLA School of Medicine, USA

**Mark Woolrich**
Oxford Centre for Human Brain
Activity, University of Oxford, UK

**Jinhu Xiong**
University of Iowa, USA

**Robert Zatorre**
MontrealNeurological Institute, Canada

**Open Access and Copyright**
All articles published by *Human Brain Mapping* submitted after January 1, 2020 are fully open access: immediately freely available to read, download, and share. All articles accepted from August 6, 2019 are published under the terms of a Creative Commons license.
Copyright on any research article published by *Human Brain Mapping* is retained by the author(s).

Further information about open access licenses and copyright can be found at: https://authorservices.wiley.com/open-research/open-access/index.html. Information on the licenses used by *Human Brain Mapping* can be found at: https://onlinelibrary.wiley.com/journal/10970193.

**Disclaimer**
The Publisher and Editors cannot be held responsible for errors or any consequences arising from the use of information contained in this journal; the views and opinions expressed do not necessarily reflect those of the Publisher and Editors, neither does the publication of advertisements constitute any endorsement by the Publisher and Editors of the products advertised. Wiley Open Access articles posted to repositories or websites are without warranty from Wiley of any kind, either express or implied, including, but not limited to, warranties of merchantability, fitness for a particular purpose, or non-infringement. To the fullest extent permitted by law Wiley disclaims all liability for any loss or damage arising out of, or in connection, with the use of or inability to use the content.

### HUMAN BRAIN MAPPING

A Journal Devoted to
Functional Neuroanatomy
and Neuroimaging

VOLUME 44, NUMBER 17, 1 DECEMBER 2023

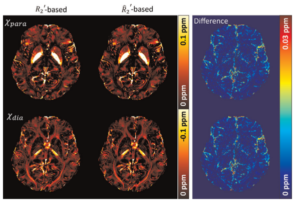

*See article by, Lao et al., pages 5953-5971, this issue*

### CONTENTS

◆ *5523* Denoising task-related fMRI: Balancing noise reduction against signal loss
**M. E. Hoeppli, M. A. Garenfeld, C. K. Mortensen, H. Nahman-Averbuch, C. D. King, and R. C. Coghill**
***Published online 27 September 2023***

◆ *5547* Semantic-specific and domain-general mechanisms for integration and update of contextual information
**Francesca M. Branzi and Matthew A. Lambon Ralph**
***Published online 3 October 2023***

◆ *5567* Spatial distribution of hand-grasp motor task activity in spinal cord functional magnetic resonance imaging
**Kimberly J. Hemmerling, Mark A. Hoggarth, Milap S. Sandhu, Todd B. Parrish, and Molly G. Bright**
***Published online 22 August 2023***

◆ *5582* Structural brain correlates of non-verbal cognitive ability in 5-year-old children: Findings from the FinnBrain birth cohort study
**Elmo P. Pulli, Saara Nolvi, Eeva Eskola, Elisabeth Nordenswan, Eeva Holmberg, Anni Copeland, Venla Kumpulainen, Eero Silver, Harri Merisaari, Jani Saunavaara, Riitta Parkkola, Tuire Lähdesmäki, Ekaterina Saukko, Eeva-Leena Kataja, Riikka Korja, Linnea Karlsson, Hasse Karlsson, and Jetro J. Tuulari**
***Published online 22 August 2023***

◆ *5602* Lifespan neurodegeneration of the human brain in multiple sclerosis
**Pierrick Coupé, Vincent Planche, Boris Mansencal, Reda A. Kamroui, Ismail Koubiyr, José V. Manjòn, and Thomas Tourdias**
***Published online 24 August 2023***

(***continued on overleaf***)

(*continued from overleaf*)

◆ *5612* Sex-dependent differences in connectivity patterns are related to episodic memory recall
**Klara Spalek, David Coynel, Dominique de Quervain, and Annette Milnik**
***Published online 30 August 2023***

◆ *5624* Multiplexity of human brain oscillations as a personal brain signature
**Stavros I. Dimitriadis, B. Routley, David E. J. Linden, and Krish D. Singh**
***Published online 5 September 2023***

◆ *5641* Evidence for adaptive myelination of subcortical shortcuts for visual motion perception in healthy adults
**Elise G. Rowe, Yubing Zhang, and Marta I. Garrido**
***Published online 22 August 2023***

◆ *5655* Healthcare experience affects pain-specific responses to others' suffering in the anterior insula
**Corrado Corradi-Dell'Acqua, Christoph Hofstetter, Gil Sharvit, Olivier Hugli, and Patrik Vuilleumier**
***Published online 22 August 2023***

◆ *5672* Unsupervised contrastive graph learning for resting-state functional MRI analysis and brain disorder detection
**Xiaochuan Wang, Ying Chu, Qianqian Wang, Liang Cao, Lishan Qiao, Limei Zhang, and Mingxia Liu**
***Published online 5 September 2023***

◆ *5693* Distributed attribute representation in the superior parietal lobe during probabilistic decision-making
**Pinchun Wang, Shuning Chen, Kun Deng, Bin Zhang, Hohjin Im, Junjiao Feng, Liqing Liu, Qinghao Yang, Guang Zhao, Qinghua He, Chunhui Chen, He Wang, and Qiang Wang**
***Published online 24 August 2023***

◆ *5712* Denoising brain networks using a fixed mathematical phase change in independent component analysis of magnitude-only fMRI data
**Chao-Ying Zhang, Qiu-Hua Lin, Yan-Wei Niu, Wei-Xing Li, Xiao-Feng Gong, Fengyu Cong, Yu-Ping Wang, and Vince D. Calhoun**
***Published online 30 August 2023***

(*continued on overleaf*)

(*continued from overleaf*)

◆ *5729* Identifying canonical and replicable multi-scale intrinsic connectivity networks in 100k+ resting-state fMRI datasets
**A. Iraji, Z. Fu, A. Faghiri, M. Duda, J. Chen, S. Rachakonda, T. DeRamus, P. Kochunov, B. M. Adhikari, A. Belger, J. M. Ford, D. H. Mathalon, G. D. Pearlson, S. G. Potkin, A. Preda, J. A. Turner, T. G. M. van Erp, J. R. Bustillo, K. Yang, K. Ishizuka, A. Faria, A. Sawa, K. Hutchison, E. A. Osuch, J. Theberge, C. Abbott, B. A. Mueller, D. Zhi, C. Zhuo, S. Liu, Y. Xu, M. Salman, J. Liu, Y. Du, J. Sui, T. Adali, and V. D. Calhoun**
***Published online 3 October 2023***

◆ *5749* Dynamic network characteristics of adolescents with major depressive disorder: Attention network mediates the association between anhedonia and attentional deficit
**Yujiao Wen, Hong Li, Yangxi Huang, Dan Qiao, Tian Ren, Lei Lei, Gaizhi Li, Chunxia Yang, Yifan Xu, Min Han, and Zhifen Liu**
***Published online 8 September 2023***

◆ *5770* Depression recurrence is accompanied by longer periods in default mode and more frequent attentional and reward processing dynamic brain-states during resting-state activity
**Sonsoles Alonso, Anna Tyborowska, Nessa Ikani, Roel J. T. Mocking, Caroline A. Figueroa, Aart H. Schene, Gustavo Deco, Morten L. Kringelbach, Joana Cabral, and Henricus G. Ruhé**
***Published online 6 September 2023***

◆ *5784* The spectral slope as a marker of excitation/inhibition ratio and cognitive functioning in multiple sclerosis
**Fahimeh Akbarian, Chiara Rossi, Lars Costers, Marie B. D'hooghe, Miguel D'haeseleer, Guy Nagels, and Jeroen Van Schependom**
***Published online 6 September 2023***

◆ *5795* Large-scale network dynamics underlying the first few hundred milliseconds after stimulus presentation: An investigation of visual recognition memory using iEEG
**Jakub Kopal, Jaroslav Hlinka, Elodie Despouy, Luc Valton, Marie Denuelle, Jean-Christophe Sol, Jonathan Curot, and Emmanuel J. Barbeau**
***Published online 9 September 2023***

◆ *5810* Both stronger and weaker cerebro-cerebellar functional connectivity patterns during processing of spoken sentences in autism spectrum disorder
**Jussi Alho, John G. Samuelsson, Sheraz Khan, Fahimeh Mamashli, Hari Bharadwaj, Ainsley Losh, Nicole M. McGuiggan, Steven Graham, Zein Nayal, Tyler K. Perrachione, Robert M. Joseph, Catherine J. Stoodley, Matti S. Hämäläinen, and Tal Kenet**
***Published online 9 September 2023***

(*continued on overleaf*)

(*continued from overleaf*)

◆ *5828* Chromatic fusion: Generative multimodal neuroimaging data fusion provides multi-informed insights into schizophrenia
**Eloy P.T. Geenjaar, Noah L. Lewis, Alex Fedorov, Lei Wu, Judith M. Ford, Adrian Preda, Sergey M. Plis, and Vince D. Calhoun**
***Published online 27 September 2023***

◆ *5846* Microstates of the cortical brain-heart axis
**Vincenzo Catrambone and Gaetano Valenza**
***Published online 9 September 2023***

◆ *5858* Impact of sample size and regression of tissue-specific signals on effective connectivity within the core default mode network
**Alexander N. Silchenko, Felix Hoffstaedter, and Simon B. Eickhoff**
***Published online 15 September 2023***

◆ *5871* A multimodal cortical network of sensory expectation violation revealed by fMRI
**Miro Grundei, Timo Torsten Schmidt, and Felix Blankenburg**
***Published online 18 September 2023***

◆ *5892* dcSBM: A federated constrained source-based morphometry approach for multivariate brain structure mapping
**Debbrata K. Saha, Rogers F. Silva, Bradley T. Baker, Rekha Saha, and Vince D. Calhoun**
***Published online 14 October 2023***

◆ *5906* Spatiotemporal dedifferentiation of the global brain signal topography along the adult lifespan
**Yujia Ao, Chengxiao Yang, Jan Drewes, Muliang Jiang, Lihui Huang, Xiujuan Jing, Georg Northoff, and Yifeng Wang**
***Published online 6 October 2023***

◆ *5919* Compressed primary-to-transmodal gradient is accompanied with subcortical alterations and linked to neurotransmitters and cellular signatures in major depressive disorder
**Yang Xiao, Lei Zhao, Xuelian Zang, and Shao-Wei Xue**
***Published online 9 September 2023***

◆ *5936* Alpha and theta band activity share information relevant to proactive and reactive control during conflict-modulated response inhibition
**Charlotte Pscherer, Paul Wendiggensen, Moritz Mückschel, Annet Bluschke, and Christian Beste**
***Published online 20 September 2023***

(*continued on overleaf*)

(*continued from overleaf*)

◆ *5953* Sub-voxel quantitative susceptibility mapping for assessing whole-brain magnetic susceptibility from ages 4 to 80
**Guoyan Lao, Qiangqiang Liu, Zhenghao Li, Xiaojun Guan, Xiaojun Xu, Yuyao Zhang, and Hongjiang Wei**
***Published online 18 September 2023***

◆ *5972* Task-dependent spatial processing in the visual cortex
**G. Bertonati, M. B. Amadeo, C. Campus, and M. Gori**
***Published online 9 October 2023***

◆ *5982* Estimation of fMRI responses related to epileptic discharges using Bayesian hierarchical modeling
**Zhengchen Cai, Nicolás von Ellenrieder, Andreas Koupparis, Hui Ming Khoo, Satoru Ikemoto, Masataka Tanaka, Chifaou Abdallah, Saba Rammal, Francois Dubeau, and Jean Gotman**
***Published online 26 September 2023***

◆ *6001* Increased microglia activation in late non-central nervous system cancer survivors links to chronic systemic symptomatology
**Poppy L. A. Schoenberg, Alexander K. Song, Emily M. Mohr, Baxter P. Rogers, Todd E. Peterson, and Barbara A. Murphy**
***Published online 26 September 2023***

◆ *6020* A metabolism-functional connectome sparse coupling method to reveal imaging markers for Alzheimer's disease based on simultaneous PET/MRI scans
**Luyao Wang, Huanyu Xu, Min Wang, Matthias Brendel, Axel Rominger, Kuangyu Shi, Ying Han, and Jiehui Jiang**
***Published online 23 September 2023***

◆ *6031* The unity and diversity of verbal and visuospatial creativity: Dynamic changes in hemispheric lateralisation
**Yixin Gao, Xinran Wu, Yuchi Yan, Min Li, Facai Qin, Mujie Ma, Xiaoning Yuan, Wenjing Yang, and Jiang Qiu**
***Published online 29 September 2023***

◆ *6043* Developmental changes in endogenous testosterone have sexually-dimorphic effects on spontaneous cortical dynamics
**Giorgia Picci, Lauren R. Ott, Samantha H. Penhale, Brittany K. Taylor, Hallie J. Johnson, Madelyn P. Willett, Hannah J. Okelberry, Yu-Ping Wang, Vince D. Calhoun, Julia M. Stephen, and Tony W. Wilson**
***Published online 9 October 2023***

(*continued on overleaf*)

(*continued from overleaf*)

◆ *6055* Reconstructing the somatotopic organization of the corticospinal tract remains a challenge for modern tractography methods
**Jianzhong He, Fan Zhang, Yiang Pan, Yuanjing Feng, Jarrett Rushmore, Erickson Torio, Yogesh Rathi, Nikos Makris, Ron Kikinis, Alexandra J. Golby, and Lauren J. O'Donnell**
***Published online 4 October 2023***

◆ *6074* Oxytocin enhances the triangular association among behavior, resting-state, and task-state functional connectivity
**Haoming Zhang, Kun Chen, Jin Bao, and Haiyan Wu**
***Published online 29 September 2023***

◆ *6090* Prosocial decision-making under time pressure: Behavioral and neural mechanisms
**Zhengjie Liu, Hailing Zhao, Yashi Xu, Jie Liu, and Fang Cui**
***Published online 29 September 2023***

◆ *6105* Group-level brain decoding with deep learning
**Richard Csaky, Mats W. J. van Es, Oiwi Parker Jones, and Mark Woolrich**
***Published online 27 September 2023***

◆ *6120* Examining the effects of prenatal alcohol exposure on performance of the sustained attention to response task in children with an FASD
**J. F. L. Pinner, W. Collishaw, M. E. Schendel, L. Flynn, F. T. Candelaria-Cook, C. M. Cerros, M. Williams, D. E. Hill, and J. M. Stephen**
***Published online 4 October 2023***

◆ *6139* Prediction of brain age using structural magnetic resonance imaging: A comparison of accuracy and test–retest reliability of publicly available software packages
**Ruben P. Dörfel, Joan M. Arenas-Gomez, Patrick M. Fisher, Melanie Ganz, Gitte M. Knudsen, Jonas E. Svensson, and Pontus Plavén-Sigray**
***Published online 16 October 2023***

◆ *6149* Distinct roles of delta- and theta-band neural tracking for sharpening and predictive coding of multi-level speech features during spoken language processing
**Guangting Mai and William S.-Y. Wang**
***Published online 11 October 2023***

(*continued on overleaf*)

(*continued from overleaf*)

◆ *6173* Neural correlates of cognitive control deficits in pediatric mild traumatic brain injury
**Harm J. van der Horn, Andrew B. Dodd, Tracey V. Wick, Cidney R. Robertson-Benta, Jessica R. McQuaid, Anne K. Hittson, Josef M. Ling, Vadim Zotev, Sephira G. Ryman, Erik B. Erhardt, John P. Phillips, Richard A. Campbell, Robert E. Sapien, and Andrew R. Mayer**
***Published online 6 October 2023***

◆ *6185* Temporal dynamics of oscillatory activity during nonlexical language decoding: Evidence from Morse code and magnetoencephalography
**Frederick Benjamin Junker, Tobias Schmidt-Wilcke, Alfons Schnitzler, and Joachim Lange**
***Published online 4 October 2023***

◆ *6198* Pre-movement event-related potentials and multivariate pattern of EEG encode action outcome prediction
**Edward Ody, Tilo Kircher, Benjamin Straube, and Yifei He**
***Published online 4 October 2023***

◆ *6214* Decreased beta-band activity in left supramarginal gyrus reflects cognitive decline: Evidence from a large clinical dataset in patients with dementia
**Hideyuki Hoshi, Momoko Kobayashi, Yoko Hirata, Keisuke Fukasawa, Sayuri Ichikawa, and Yoshihito Shigihara**
***Published online 4 October 2023***

◆ *6227* Neural correlates of temporal recalibration to delayed auditory feedback of active and passive movements
**Christina V. Schmitter, Konstantin Kufer, Olaf Steinsträter, Jens Sommer, Tilo Kircher, and Benjamin Straube**
***Published online 11 October 2023***

◆ *6245* Aberrant degree centrality profiles during rumination in major depressive disorder
**Feng-Nan Jia, Xiao Chen, Xiang-Dong Du, Zhen Tang, Xiao-Yun Ma, Tian-Tian Ning, Si-Yun Zou, Shang-Fu Zuo, Hui-Xian Li, Shi-Xian Cui, Zhao-Yu Deng, Jia-Lin Fu, Xiao-Qian Fu, Yue-Xiang Huang, Xue-Ying Li, Tao Lian, Yi-Fan Liao, Li-Li Liu, Bin Lu, Yan Wang, Yu-Wei Wang, Zi-Han Wang, Gang Ye, Xin-Zhu Zhang, Hong-Liang Zhu, Chuan-Sheng Quan, Hong-Yan Sun, Chao-Gan Yan, and Yan-Song Liu**
***Published online 14 October 2023***

(*continued on overleaf*)

(*continued from overleaf*)

◆ *6258* Somatotopic disruption of the functional connectivity of the primary sensorimotor cortex in complex regional pain syndrome type 1
**Jaakko Hotta, Jukka Saari, Hanna Harno, Eija Kalso, Nina Forss, and Riitta Hari**
***Published online 14 October 2023***

◆ *6275* Are we really targeting and stimulating DLPFC by placing transcranial electrical stimulation (tES) electrodes over F3/F4?
**Ghazaleh Soleimani, Rayus Kuplicki, Jazmin Camchong, Alexander Opitz, Martin P. Paulus, Kelvin O. Lim, and Hamed Ekhtiari**
***Published online 26 September 2023***
